# Fluid Shear Stress and Fibroblast Growth Factor-2 Increase Endothelial Cell-Associated Vitronectin

**DOI:** 10.1155/2017/9040161

**Published:** 2017-06-01

**Authors:** Justin G. Mathew, Sarah Basehore, Alisa Morss Clyne

**Affiliations:** ^1^School of Biomedical Engineering, Science and Health Systems, Drexel University, 3141 Chestnut Street, Philadelphia, PA 19104, USA; ^2^Mechanical Engineering and Mechanics, Drexel University, 3141 Chestnut Street, Philadelphia, PA 19104, USA

## Abstract

Vitronectin is a matricellular protein that plays an important role in both coagulation and angiogenesis through its effects on cell adhesion and the plasminogen system. Vitronectin is known to bind to endothelial cells upon integrin activation. However, the effect of integrin activation by shear stress and growth factors on cell-associated vitronectin and plasminogen system activity has not yet been studied. We therefore exposed human umbilical vein endothelial cells to steady laminar flow, oscillating disturbed flow, or fibroblast growth factor-2 (FGF-2) for 24 hours. We then measured cell-associated vitronectin by Western blot and plasminogen system activity using a Chromozym assay. Steady laminar flow, oscillating disturbed flow, and FGF-2 all increased cell-associated vitronectin, although the vitronectin molecular weight varied among the different conditions. FGF-2 also increased cell-associated vitronectin in microvascular endothelial cells and vascular smooth muscle cells. The increase in cell-associated vitronectin increased plasminogen system activity. Confocal microscopy showed that vitronectin was primarily located in the basal and intracellular regions. *α*_v_*β*_5_ integrin inhibition via genistein, an anti-*α*_v_*β*_5_ antibody, or *β*_5_ siRNA knockdown abrogated the FGF-2-induced increase in cell-associated vitronectin and increased plasminogen system activity. These data show that shear stress and growth factors increase cell-associated vitronectin through integrin activation, which may affect coagulation and angiogenesis.

## 1. Introduction

Vitronectin, a 75–79 kDa adhesive glycoprotein primarily produced by the liver, modulates cell interactions with the extracellular matrix (ECM) during cell proliferation, migration, invasion, and remodelling [1]. Vitronectin is found in the systemic circulation at a concentration of 0.2–0.4 mg/ml, and limited amounts of vitronectin are deposited in the vascular wall either associated with the endothelial cell surface or bound to the vascular ECM [1]. In the healthy vasculature, vitronectin helps regulate hemostasis, coagulation, vascular remodelling, and angiogenesis [2–4]. In disease states, vitronectin is deposited at locations of injury, inflammation, and repair where it modulates cell adhesion and migration into the damaged site [5]. The majority of these vitronectin deposits occur through receptor-mediated vitronectin uptake and extracellular matrix deposition, rather than local production [6, 7].

Increased tissue vitronectin is observed in diseases of altered blood flow, including atherosclerosis, myocardial infarction, and cancer [8–10]. Atherosclerotic plaques in particular initiate with endothelial cell dysfunction partially due to irregular blood flow [11]. In straight arterial sections, endothelial cells are exposed to steady laminar flow, leading to high, directed shear stress (10 to 70 dynes/cm^2^ in arteries) [12]. Under these conditions, endothelial cells align, elongate, and organize actin stress fibers parallel to the flow direction [13, 14]. These endothelial cells also express an atheroprotective phenotype, maintaining vascular homeostasis through tight control of permeability, inflammation, vascular tone, and injury repair [15]. At arterial branches and bends, endothelial cells are exposed to disturbed flow, which includes flow recirculation, separation, and reversal. Disturbed flow is associated with low or reciprocating shear stress, which contributes to an atheroprone endothelial phenotype [16–18]. Endothelial cells exposed to disturbed flow are round with actin filaments concentrated in the cell periphery [19, 20], proliferative [20, 21], permeable [22–24], and inflamed [25, 26].

Shear stress is known to induce endothelial cell intracellular signalling through integrin activation, which is an important mechanism through which vitronectin regulates vascular remodelling. Vitronectin binds to activated *α*_v_*β*_3_, *α*_v_*β*_5_, and *α*_v_*β*_1_ integrins on the cell membrane through its RGD motif [27]. Integrin binding is typically associated with cell adhesion, proliferation, and migration [28]. However, the *α*_v_*β*_5_ integrin in particular participates in vitronectin endocytosis [6, 7]. Once vitronectin is endocytosed, it can be either degraded or deposited in the extracellular matrix. Vitronectin also regulates vascular remodelling through the plasminogen-activating system, which promotes proteolysis. The vitronectin somatomedin B binds to plasminogen activator inhibitor (PAI-1) to stabilize PAI-1 in its active conformation and prolong its functional half-life [29, 30]. PAI-1 bound to vitronectin decreases plasminogen system activity and thereby reduces or postpones the fibrinolysis essential for blood clot resolution and ECM proteolysis essential for vascular remodelling and angiogenesis [4, 31–33].

Vitronectin plays a key role in vascular remodelling through both the plasminogen-activating system and cell integrin interactions. It is therefore important to understand how both biochemical and biomechanical stimuli impact vitronectin deposition, in particular for clinical therapies focused on enhancing or inhibiting angiogenesis. We hypothesized that shear stress would activate integrins and increase endothelial monolayer-associated vitronectin. We therefore measured endothelial cell-associated vitronectin in cells exposed to steady laminar and oscillating disturbed flow, as well as to fibroblast growth factor-2 (FGF-2) which also activates endothelial integrins. We further determined whether endothelial-associated vitronectin affected plasminogen system activity using the Chromozym PL assay. We now show that both fluid shear stress and FGF-2 increase endothelial-associated vitronectin.

## 2. Materials and Methods

### 2.1. Cell Culture

Human umbilical vein endothelial cells (HUVEC, passages 4–9; Lonza) were maintained in an endothelial growth medium (EGM-2; Lonza) supplemented with 5% fetal bovine serum (FBS; Hyclone), 1% penicillin-streptomycin (Gibco) and 1% glutamine (Gibco). HUVEC were selected because they are widely used for in vitro endothelial cell studies, show a robust shear stress and FGF-2 response, and react with human plasminogen system proteins and antibodies. Bovine brain microvascular endothelial cells (BBmVEC, passages 5–9; Cell Applications) were maintained in Dulbecco's modification of Eagle's medium (DMEM, CellGro) supplemented with 10% FBS, 1% penicillin-streptomycin, 3 ng/ml FGF-2 (Peprotech), and 3 *μ*g/ml heparin (Baker). Primary porcine aortic endothelial cells (PAEC, passages 5–9, isolated by the collagenase dispersion method) were maintained in DMEM supplemented with 5% FBS, 1% penicillin-streptomycin, and 1% glutamine [34]. Primary porcine vascular smooth muscle cells (PSMC, passages 5–9, isolated by the explant technique) were maintained in DMEM supplemented with 10% FBS, 1% penicillin-streptomycin, and 1% glutamine [35].

For shear stress experiments, HUVEC were seeded at 17,000 cells/cm^2^ on 60 × 15 mm culture dishes for 48 hours prior to testing. Cells were exposed to 24 hours of steady laminar flow (20 dynes/cm^2^ shear stress) or oscillating disturbed flow (4 ± 6 dynes/cm^2^ shear stress, 1 Hz) in a cone and plate device. For FGF-2 experiments, HUVEC were seeded at 25,000 cells/cm^2^ in the either endothelial basal medium (EBM-2, Lonza, HUVEC) or DMEM (BBmVEC, PAEC, PSMC) supplemented with FBS and allowed to attach for 48 hours. EBM-2, which is EGM-2 without growth factors or cytokines, was used for all FGF-2 experiments to prevent artifacts from growth factors in the growth medium. Samples were then treated with 50 ng/ml FGF-2 for 0–72 hours. While lower FGF-2 concentrations (e.g., 10 ng/ml) also increased plasminogen activity, a higher concentration was used to produce repeatable results.

Multimeric vitronectin (mVTN) was prepared as described [36]. Briefly, 1 mg/ml native vitronectin (nVTN, Molecular Innovations) was incubated with 6M urea (Sigma) in 1× Tris-buffered saline (TBS) for 1 hour at 37°C. After dialysis in 1× TBS for 18 hours, mVTN was collected and stored at −80°C until use.

### 2.2. Cell-Associated Vitronectin: Western Blot

Cell-associated vitronectin was quantified by Western blot. HUVEC, BBmVEC, PAEC, and PSMC samples were scraped off the surface in ice-cold lysis buffer (20 mM Tris, 150 mM NaCl, 1% Triton X-100, 0.1% SDS, 2 mM EDTA, 2 mM NaVO_4_, 2 mM PMSF, 50 mM NaF, 10% glycerol, complete protease inhibitor (Roche), pH 7.4). Cell lysates were normalized for protein content, separated by SDS-PAGE on a 4–12% Bis-Tris gel (Life Technologies), and transferred to nitrocellulose membranes using the Invitrogen iBlot system. Membranes were incubated overnight at 4°C with vitronectin primary antibody (sc-15332, Santa Cruz), followed by an anti-rabbit secondary horseradish peroxidase-conjugated antibody (Promega) for 2 hours at room temperature. Protein bands were detected using an enhanced chemiluminescence kit (Western Lightning, PerkinElmer) and visualized with a Fluorchem digital imager (Alpha Innotech). Both native and multimeric vitronectin exhibited a 65/75 kDa doublet under reducing conditions, as previously reported [37]. Doublet intensity was quantified using AlphaEase FC software.

### 2.3. Vitronectin Expression: RT-PCR

Reverse transcription-polymerase chain reaction (RT-PCR) was used to determine if HUVEC produce vitronectin in vitro. HUVEC were cultured as described, and mRNA was isolated using the PureLink RNA mini kit (Ambion) as per manufacturer protocol. A cDNA library was created by combining 500 ng isolated mRNA with 50 *μ*M oligo(dT)_20_ primers and annealing buffer in a 0.2 ml thin-walled PCR tube. The sample was then incubated in a thermal cycler at 65°C for 5 minutes and placed on ice for 1 minute. Contents were collected and briefly centrifuged. 2X First-Strand Reaction Mix and SuperScript III/RNaseOUT enzyme mix (Life Technologies) were combined and added to the tube. The mixture was incubated in a thermal cycler for 50 min at 50°C. Reaction was terminated at 85°C for 5 min. cDNA was stored at −20°C. PCR was performed by preheating the thermal cycler to 95°C and preparing master mix: 10 *μ*M forward primer, 10 *μ*M reverse primer, and Taq 2X Master Mix (New England BioLabs) in nuclease-free water. 5 *μ*l cDNA was then added into the reaction tube. The thermal cycler settings were as follows: initial denaturation at 95°C for 30 seconds; 30 cycles at 95°C for 30 seconds, 65°C for 30 seconds, and 68°C for 1 minute; and final extension at 68°C for 5 minutes and held at 4–10°C. Amplicons were stored at −20°C. Samples were then combined with TBE Hi-Density Sample Buffer (5×) (Life Technologies) and diH_2_O and loaded onto a 6% TBE polyacrylamide gel (Life Technologies). The gel was run at 200 V for 120 min with an expected current of 10–18 mA/gel (start) and 4–6 mA/gel (end). The gel was stained with 0.5 *μ*g/ml ethidium bromide (Biorad) by soaking for 15 minutes with gentle agitation. The gel was rinsed and then imaged using a 302 nm UV transilluminator, and protein bands were quantified by ImageJ.

### 2.4. Vitronectin Location: Confocal Microscopy

Vitronectin location was determined by confocal microscopy. HUVEC were cultured and treated with FGF-2 for 24 hours as described. After FGF-2 treatment, cells were rinsed, fixed with 4% paraformaldehyde for 20 minutes at room temperature, and thoroughly washed in PBS. Samples were then permeabilized with 0.1% Triton X-100 in PBS for 4 minutes at room temperature. After thorough washing and blocking for 30 minutes with 1% BSA in PBS, samples were incubated with the vitronectin primary antibody at room temperature for 30 minutes in 0.1% BSA. Samples were then incubated with an AlexaFluor 488 secondary antibody (1 : 200, Life Technologies) and Hoechst (1 : 2000, Life Technologies) for 1 hour at room temperature. After thorough washing, samples were imaged in a z-stack (1 *μ*m depth, 12 images per sample) using an Olympus IX81 inverted confocal microscope. Mean intensity values for basal, intracellular, or apical sections as well as for the entire image stack were quantified with ImageJ.

### 2.5. Plasminogen System Activity: Chromozym PL

Chromozym PL was used to determine plasminogen system activity in cell extracts. In this assay, uPA in the cell extract cleaves exogenous plasminogen to plasmin, which then cleaves Chromozym PL into a residual peptide and 4-nitroaniline (405 nm). HUVEC were cultured and treated with FGF-2 as described. Cells were then lysed in T/T buffer (60 mM Tris hydrochloride, 0.5% Triton X-100) for 5 minutes, after which cell extracts were centrifuged at 10,000*g* for 10 minutes to remove insoluble material. A final solution of cell extract, 127 ng/ml Chromozym PL (Roche), and 67 *μ*U/ml plasminogen (Roche) was mixed in a 96-well plate. Absorbance (405 nm) was measured for 24 hours in an Infinite 200 PRO microplate reader (TECAN) maintained at 37°C. The change in 4-nitroaniline absorbance at 405 nm is directly proportional to uPA enzymatic activity. Absorbance was plotted versus time, and the linear region slope (Δ*A*/min) was used to calculate plasmin activity via the following equation:

(1) $$\begin{array}{c} plasmin\;activity\;\left(\frac{U}{ml}\right)=\left[\left(\left(\frac{V}{v}\right)*\in *d\right)*\left(\frac{\Delta A}{\min}\right)\right], \end{array}$$

where *V* is the total volume (300 *μ*l), *v* is the cell extract volume (33 *μ*l), *∈* is the absorbance coefficient for 4-nitroaniline (10.4 mmol^−1^ · cm^−1^), and *d* is the light path (1 cm).

### 2.6. Integrin Inhibition: Genistein, Blocking Antibody, and *β*_5_ siRNA Transfection

The role of FGF-2-induced integrin activation in the increase in cell-associated vitronectin was assessed using genistein, integrin-blocking antibodies, and *β*_5_ siRNA. To inhibit integrin tyrosine phosphorylation, cells were pretreated with 30 *μ*g/ml of the protein tyrosine kinase inhibitor genistein (Sigma) for 2 hours in supplemented EBM-2. Genistein was then removed, and cells were treated with FGF-2. To block vitronectin binding to the *α*_v_*β*_5_ integrin, HUVEC were incubated with 1 *μ*g/ml anti-*α*_v_*β*_5_ antibody (Millipore) concurrent with FGF-2 treatment. To downregulate the *α*_v_*β*_5_ integrin, HUVEC were seeded at 60,000 cells/well in 1 ml supplemented medium and cultured at 37°C for 24 hours. Lipofectamine (3 *μ*l/well) and 10 *μ*M *β*_5_ siRNA were added to each sample for 72 hours. Decreased *β*_5_ was confirmed by Western blot.

### 2.7. Statistical Analysis

Statistical analysis was performed with GraphPad Prism and Instat software. Samples were collected in triplicate, and experiments were performed at least two times. Data are graphed as mean ± standard deviation. Significance between two groups was compared using Student's *t*-test. Comparisons among multiple groups were analyzed by two-way ANOVA with a Bonferroni post hoc test. *p* values are indicated in the figures by #*p* < 0.05, ^∗^*p* < 0.01, and ^∗∗^*p* < 0.001, unless otherwise indicated.

## 3. Results

### 3.1. Endothelial Cell-Associated Vitronectin Increased in Cells Exposed to Fluid Shear Stress and FGF-2

**Figure 1:**
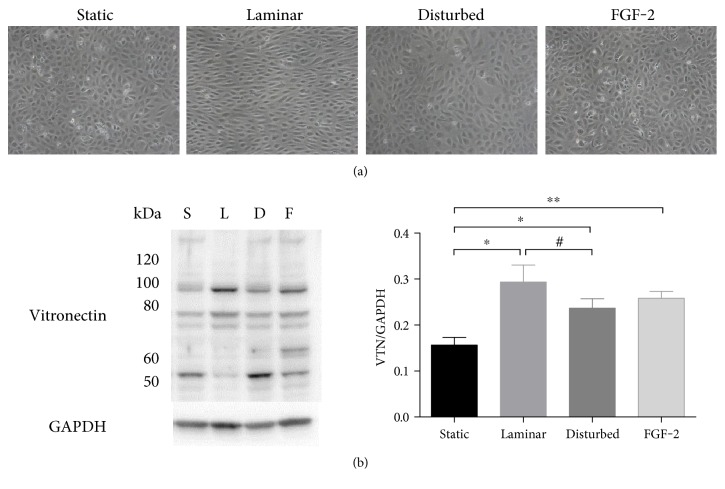
Both shear stress and FGF-2 increased cell-associated vitronectin. HUVECs were exposed to steady laminar flow (20 dynes/cm^2^) or oscillating disturbed flow (4 ± 6 dynes/cm^2^ shear stress, 1 Hz) in a cone and plate device, or 50 ng/ml FGF-2 for 24 hours. Samples were then analyzed for vitronectin by Western blot. (a) HUVEC phase contrast microscopy images following 24 hours of flow or FGF-2 (10x). (b) Vitronectin (VTN) Western blot (one sample out of three replicates), with GAPDH loading control and quantification of the 75/65 kDa band (normalized to GAPDH). ^∗^*p* < 0.05, ^∗∗^*p* < 0.01. One representative experiment of two.

We first determined if 24 hours of exposure to three conditions that activate integrins (steady laminar flow, oscillating disturbed flow, FGF-2) increased cell-associated vitronectin in HUVEC monolayers. HUVEC appeared aligned and elongated after steady laminar flow, while cells in the other three conditions remained polygonal (Figure 1(a)). Cells exposed to steady laminar flow also had increase phosphorylated endothelial nitric oxide synthase, which confirmed that they responded to the applied shear stress. Cell death (by Live/Dead assay) and proliferation (by Ki67 labeling) were low in all samples, but cells adapted to steady laminar flow had essentially no dying or proliferating cells (data not shown). When cell samples were probed for vitronectin by Western blot, all treatment conditions showed a statistically significant increase in total cell-associated vitronectin (Figure 1(b)). However, the distribution of vitronectin molecular weight varied by sample. Endothelial cells exposed to steady laminar flow showed more vitronectin at ~90 kDa and less vitronectin at ~50 kDa than cells in static conditions, oscillating disturbed flow, or FGF-2. Since all integrin-activating conditions increased the vitronectin doublet at 75/65 kDa, and these two bands represent the primary molecular forms of vitronectin, we analyzed vitronectin at these molecular weights for all subsequent experiments.

### 3.2. FGF-2 Increased Cell-Associated Vitronectin in Varied Vascular Cell Types

**Figure 2:**
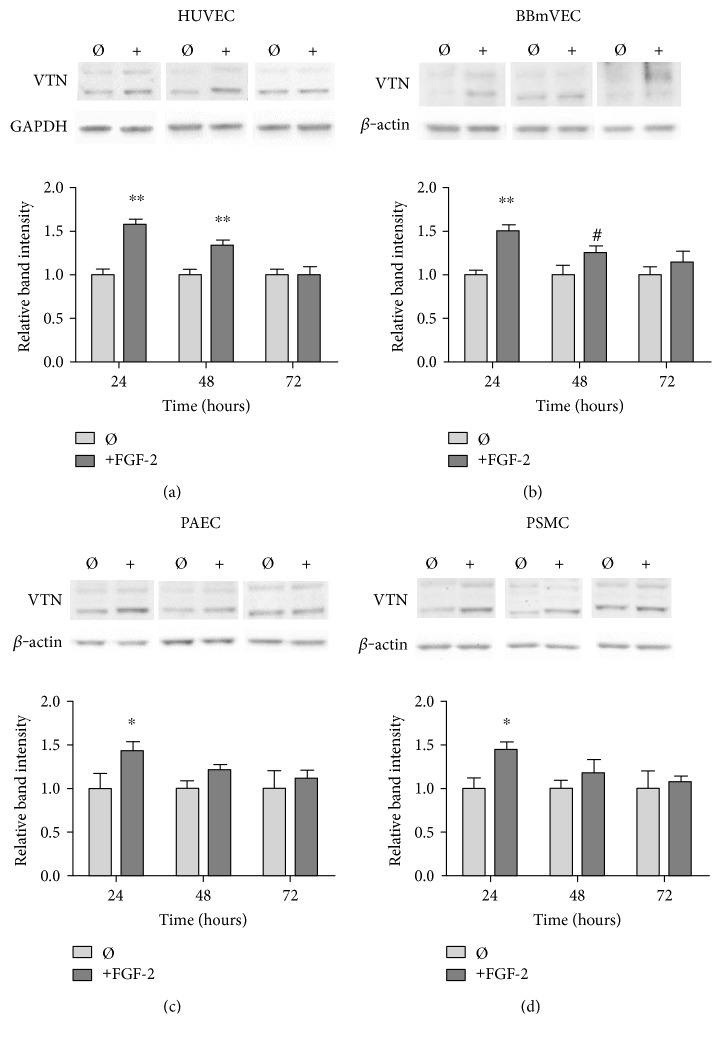
FGF-2 increased cell-associated vitronectin in (a) HUVEC (human umbilical vein endothelial cells), (b) BBmVEC (bovine brain microvascular endothelial cells), (c) PAEC (porcine aortic endothelial cells), and (d) PSMC (porcine vascular smooth muscle cells). Cells seeded on native collagen-coated substrates were stimulated with 50 ng/ml FGF-2 for 24, 48, and 72 hours. Cell extracts were collected, and normalized protein samples were analyzed by Western blot. VTN intensity was quantified and normalized to GAPDH or *β*-actin and then normalized to untreated cell VTN levels at 24 hours. ^#^*p* < 0.05, ^∗^*p* < 0.01, and ^∗∗^*p* < 0.001 compared to those of the untreated cells. *n* = 3, one representative experiment of two.

To determine the specificity of this effect, we measured cell-associated vitronectin over time in endothelial cells from different vascular beds and alternative species, as well as in vascular smooth muscle cells. Since not all cell types respond to shear stress, we focused on FGF-2 treatment. No significant changes were observed in any cell type with 12 hours of FGF-2 treatment (data not shown). In HUVEC, FGF-2 increased cell-associated vitronectin by 58% as compared to untreated cells after 24 hours. Cell-associated vitronectin then decreased at 48 hours and returned to control levels by 72 hours (Figure 2(a)). Cell-associated vitronectin was similarly elevated by 51% after 24 hours of FGF-2 treatment in bovine brain microvascular endothelial cells (BBmVEC, Figure 2(b)); by 43% in porcine aortic endothelial cells (PAEC, Figure 2(c)); and by 45% in porcine smooth muscle cells (PSMC, Figure 2(d)). A two-way ANOVA showed that FGF-2 and time, as well as their interaction, were statistically significant (*p* < 0.001) in all cell types. These data indicate that FGF-2 increased cell-associated vitronectin in animal and human macro- and microvascular endothelial and smooth muscle cells in a time-dependent manner.

### 3.3. Endothelial Cell-Associated Vitronectin with FGF-2 Came from Serum

**Figure 3:**
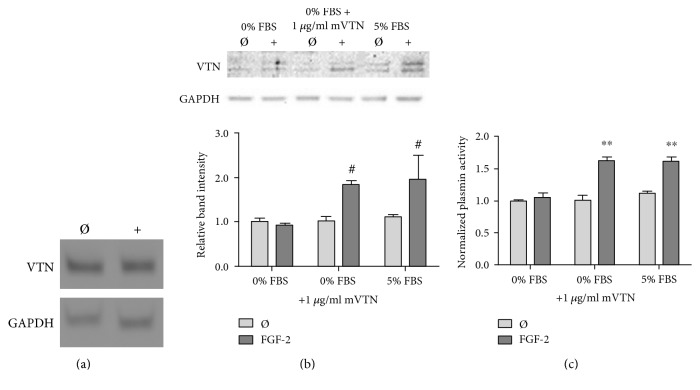
HUVEC produced vitronectin mRNA, but the FGF-2-induced increased in cell-associated vitronectin which came from the serum. (a) mRNA was isolated via reverse transcriptase PCR in HUVEC which were treated with 50 ng/ml FGF-2 for 24 hours. (b) Cell-associated vitronectin was analyzed by Western blot in HUVEC treated with 50 ng/ml FGF-2 for 24 hours in EBM-2 0% FBS, 1 *μ*g/ml mVTN, or 5% FBS. Vitronectin band intensity was quantified and normalized to GAPDH and then normalized to vitronectin levels in untreated cells in the serum-free medium. (c) Plasmin enzymatic activity was analyzed in HUVEC treated with 50 ng/ml FGF-2 for 24 hours in EBM-2 0% FBS, 1 *μ*g/ml mVTN, or 5% FBS by the Chromozym assay. All samples were normalized to untreated cells in the serum-free medium. ^#^*p* < 0.05 and ^∗∗^*p* < 0.001 compared to those of the untreated cells in the serum-free medium. *n* = 3, one representative experiment of three.

Vitronectin is primarily produced by hepatocytes and circulated throughout the body in serum; however, some cell types produce vitronectin at low levels in vivo and in vitro [1]. We therefore measured HUVEC vitronectin mRNA to assess production. Through PCR, we qualitatively showed that in fact HUVEC did produce vitronectin mRNA both with and without FGF-2 stimulation (Figure 3(a)). To determine whether cell-associated vitronectin came from HUVEC vitronectin production, we measured the effect of FGF-2 treatment on cell-associated vitronectin in a serum-free medium. HUVEC treated with FGF-2 in the serum-free medium showed no change in cell-associated vitronectin (Figure 3(b)). However, when 1 *μ*g/ml mVTN was added into the serum-free medium, cell-associated vitronectin increased 85% with FGF-2 treatment. This was statistically similar to cells in medium with 5% serum. We then assessed functional response to cell-associated vitronectin by examining plasmin activity using the Chromozym assay. Similarly, FGF-2 stimulation did not increase plasminogen system activity for cells in the serum-free medium (Figure 3(c)). When mVTN was returned to the system, plasminogen system activity increased by nearly 60% with FGF-2 treatment. These data suggest the cell-associated vitronectin came from exogenous sources (serum or added vitronectin) rather than from HUVEC production. Since FGF-2 effects in the serum-free medium with added mVTN were statistically similar to FGF-2 effects in medium with 5% serum, all subsequent experiments were performed in medium with serum.

### 3.4. FGF-2 Increased Cell-Associated Basal and Intracellular Vitronectin

**Figure 4:**
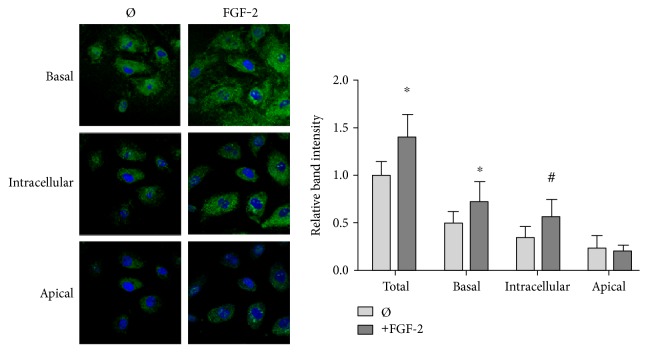
FGF-2 stimulation increased basal and intracellular vitronectin. Confocal microscopy images of cells labeled for vitronectin (AF488, green) and nuclei with (Hoescht, blue). HUVEC treated with 50 ng/ml FGF-2 for 24 hours were permeabilized with Triton X-100 to label membrane and cytoplasmic vitronectin. Samples were imaged in a z-stack (1 *μ*m depth, 12 images per sample) to obtain fluorescence intensity throughout the cell. Images in the z-stack were classified as basal (below the nucleus), intracellular (at the same levels as the nucleus), and apical (above the nucleus). Mean intensity values were quantified with ImageJ. All samples were normalized to total vitronectin in untreated cells. ^#^*p* < 0.05 versus that of the untreated cells. *n* = 3, one representative experiment of three.

Vitronectin can bind to *α*_v_*β*_3_ or *α*_v_*β*_5_ integrins, where it can either create cell-surface complexes or be endocytosed [7, 38]. We used confocal microscopy to determine whether FGF-2 caused vitronectin to bind to the cell surface and then remain on the apical side of the endothelium or whether FGF-2 caused vitronectin to be either endo- or transcytosed. After treatment with FGF-2 for 24 hours, HUVEC were labeled for vitronectin and imaged by confocal microscopy (Figure 4). Fluorescence intensity in the z-stacks (1 *μ*m depth, 12 images per sample) was quantified after they were divided into basal, intracellular, and apical sections based on whether the images were below, at the same level as, or above the cell nucleus, respectively. FGF-2 increased basal and intracellular vitronectin labeling by around 50% but did not increase apical vitronectin. These data suggest that FGF-2 increased endothelial cell vitronectin endo- or transcytosis.

### 3.5. Cell-Associated Vitronectin Was Inhibited by Preventing *α*_v_*β*_5_ Integrin Activation

**Figure 5:**
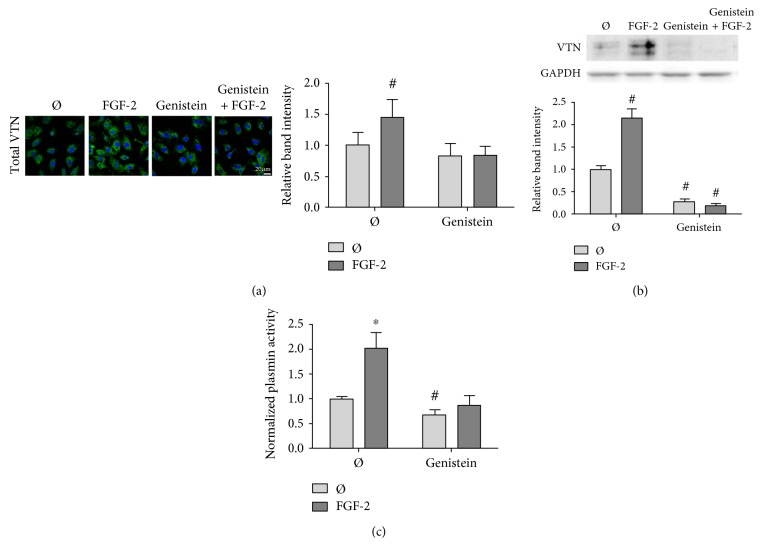
FGF-2-induced cell-associated vitronectin and plasminogen system activity were abrogated by genistein, a protein tyrosine kinase inhibitor. HUVEC were exposed to 30 *μ*g/ml genistein for 2 hours prior to stimulation with 50 ng/ml FGF-2 for 24 hours. (a) Samples were fixed and permeabilized with Triton X-100 to visualize total vitronectin. Samples were then labeled for vitronectin (green) and nuclei (blue). Samples were imaged in a z-stack (1 *μ*M depth, 12 images per sample) to obtain fluorescence intensity throughout the cell. Mean intensity values were quantified with ImageJ. (b) Cell-associated vitronectin was analyzed by Western blot in cells treated with FGF-2 and genistein. Vitronectin band intensity was quantified and normalized to GAPDH and then normalized to untreated cell levels. (c) Plasmin enzymatic activity was determined using the Chromozym assay in HUVEC treated with FGF-2 and genistein. Samples were normalized to untreated cells. ^#^*p* < 0.05 and ^∗^*p* < 0.01 compared to those of the untreated cells. *n* = 3, one representative experiment of two.

Both fluid shear stress and growth factors activate integrins [39, 40], and these activated integrins then bind to vitronectin [41]. To determine if FGF-2 increased cell-associated vitronectin via integrin activation, we first blocked integrin tyrosine phosphorylation in FGF-2-treated HUVEC using the protein tyrosine kinase inhibitor genistein [42]. By both confocal microscopy (Figure 5(a)) and Western blot (Figure 5(b)), genistein abrogated the FGF-2-induced increase in endothelial cell-associated vitronectin. As a test of the functional effect of tyrosine kinase inhibition, genistein further abolished the increase in plasminogen system activity due to FGF-2 stimulation (Figure 5(c)).

**Figure 6:**
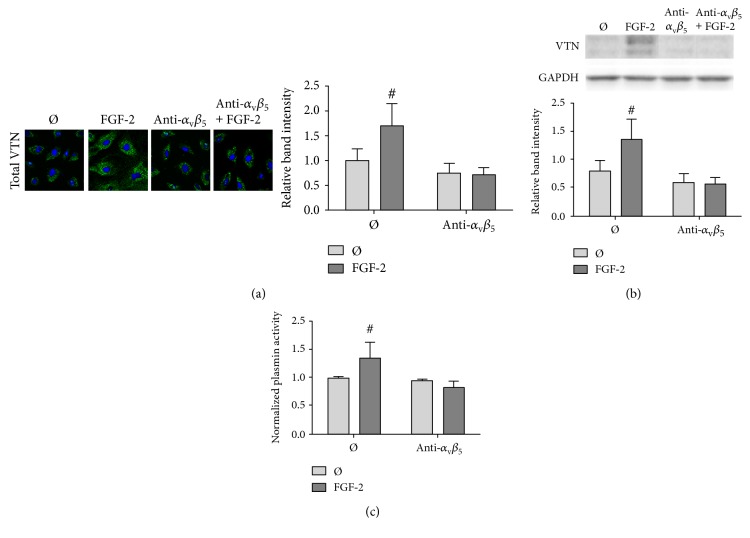
FGF-2-induced cell-associated vitronectin and plasminogen system activity were abrogated when the *α*_v_*β*_5_ integrin was blocked. HUVEC were treated with 1 *μ*g/ml anti-*α*_v_*β*_5_-blocking antibody concurrently with 50 ng/ml FGF-2 for 24 hours. (a) Samples were fixed and permeabilized with Triton X-100 to visualize total vitronectin. Samples were then labeled for vitronectin (green) and nuclei (blue). Samples were imaged in a z-stack (1 *μ*M depth, 12 images per sample) to obtain fluorescence intensity throughout the cell. Mean intensity values were quantified with ImageJ. (b) Cell-associated vitronectin was analyzed by Western blot in cells treated with FGF-2 and genistein. Vitronectin band intensity was quantified and normalized to GAPDH and then normalized to untreated cell levels. (c) Plasmin enzymatic activity was determined using the Chromozym assay in HUVEC treated with FGF-2 and genistein. Samples were normalized to untreated cells. ^#^*p* < 0.05 and ^∗^*p* < 0.01 compared to those of the untreated cells. *n* = 3, one representative experiment of two.

Since genistein is a general tyrosine phosphorylation inhibitor, we used a more integrin-specific approach by blocking the *α*_v_*β*_5_ integrin using a neutralizing antibody. We then assessed the role of integrin activation in the FGF-2-induced increase in cell-associated vitronectin. Again, by both confocal microscopy (Figure 6(a)) and Western blot (Figure 6(b)), the anti-*α*_v_*β*_5_ integrin neutralizing antibody blocked the increase in HUVEC-associated vitronectin with FGF-2 stimulation. The integrin-neutralizing antibody further abrogated the FGF-2-induced increase in plasminogen system activity (Figure 6(c)).

**Figure 7:**
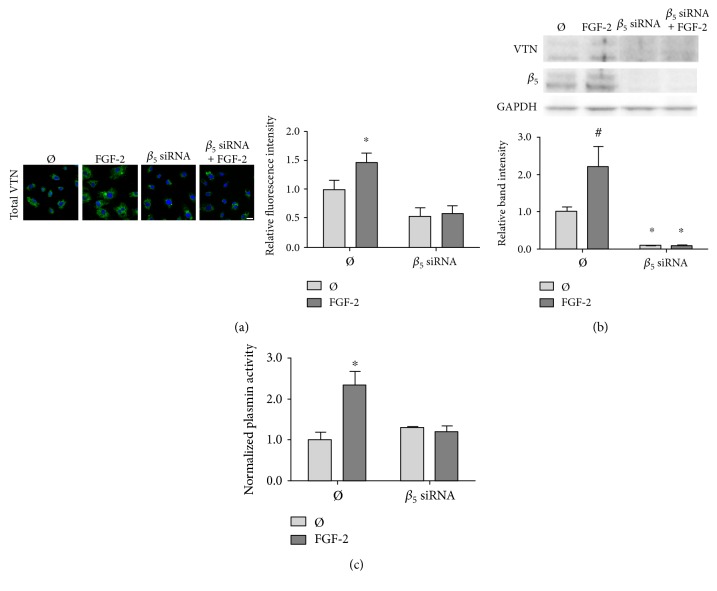
FGF-2-induced cell-associated vitronectin and plasminogen system activity were abrogated by silencing the *β*_5_ integrin with siRNA. HUVEC were transfected with *β*_5_ siRNA for 72 hours. Samples were then treated with 50 ng/ml FGF-2 for 24 hours. (a) Samples were fixed and permeabilized with Triton X-100 to visualize total vitronectin. Samples were then labeled for vitronectin (green) and nuclei (blue). Samples were imaged in a z-stack (1 *μ*M depth, 12 images per sample) to obtain fluorescence intensity throughout the cell. Mean intensity values were quantified with ImageJ. (b) Cell-associated vitronectin was analyzed by Western blot in cells treated with FGF-2 and genistein. Vitronectin band intensity was quantified and normalized to GAPDH and then normalized to untreated cell levels. (c) Plasmin enzymatic activity was determined using the Chromozym assay in HUVEC treated with FGF-2 and genistein. Samples were normalized to untreated cells. ^#^*p* < 0.05 and ^∗^*p* < 0.01 compared to those of the untreated cells. *n* = 3, one representative experiment of two.

To confirm the role of the *α*_v_*β*_5_ integrin, we silenced the *α*_v_*β*_5_ integrin using *β*_5_ siRNA to remove any integrin activation by FGF-2. We then measured cell-associated vitronectin after FGF-2 treatment by confocal microscopy and Western blot. *β*_5_ siRNA knockdown completely abolished FGF-2-induced cell-associated vitronectin, as measured by both confocal microscopy (Figure 7(a)) and Western blot (Figure 7(b)). *β*_5_ siRNA knockdown also eliminated FGF-2-induced plasminogen system activity (Figure 7(c)).

## 4. Discussion

Vitronectin is deposited in areas of altered blood flow in vascular disease in vivo; however, the effect of fluid shear stress and integrin activation on cell-associated vitronectin was unknown. We now show for the first time that steady laminar flow, oscillating disturbed flow, and FGF-2 increase endothelial cell-associated vitronectin. The amount of vitronectin peaks at 24 hours in varied vascular cell types and then decays to baseline by 72 hours. Since both flow and FGF-2 activate cell integrins, we believe that the common pathway for the increase in cell-associated vitronectin with both stimuli is integrin activation. Despite similarities, there were important differences in cell-associated vitronectin molecular weight with each stimulus. We further demonstrated that for FGF-2-treated cells, cell-associated vitronectin comes from the serum and impacts plasminogen system activity. Thus, both shear stress and FGF-2 may alter vascular remodelling through vitronectin-mediated effects on plasminogen-driven matrix degradation.

The difference in cell-associated vitronectin molecular weight in endothelial cells exposed to steady laminar flow, oscillating disturbed flow, and FGF-2 is particularly interesting. The different molecular weights likely represent vitronectin multimers and degradation fragments. Much of vitronectin's activity depends on its conformational state. More than 95% of circulating vitronectin is in its native form, with a folded conformation and therefore limited ligand-binding activity [43]. However, when native vitronectin is denatured by PAI-1, thrombin, or heparin, vitronectin assumes a multimeric form [44]. Multimeric vitronectin then has enhanced interaction with PAI-1 as well as cell surface receptors (such as integrins) and the ECM [45, 46]. We performed exploratory experiments to determine if we could alter vitronectin multimers or fragments in endothelial cells adapted to steady laminar flow using PAI-1; however, PAI-1 did not change the vitronectin Western blot for any tested condition. Additional research is needed to determine how laminar flow alters vitronectin multimerization and/or cleavage, as well as the physiological significance of these changes.

In all of the vascular cell types that we tested, cell-associated vitronectin peaked at 24 hours and then decreased to baseline levels by 72 hours. The decay likely relates to vitronectin endocytosis and degradation. After binding to the cell surface, vitronectin is processed by the cell through endo- or transcytosis for degradation or ECM deposition, respectively [1]. In fibroblasts, multimeric vitronectin internalization and subsequent degradation was inhibited by antibodies against the *α*_v_*β*_5_ integrin but not the *α*_v_*β*_3_ integrin [6, 37]. Thus, endothelial cell integrin activation from flow or FGF-2 may also promote vitronectin endocytosis and degradation. The decay in cell-associated vitronectin after 24 hours could result from other processes that are initially stimulated by flow or FGF-2 but take several days to develop. For example, the glycocalyx and adherens junctions are both critical to endothelial barrier function [47, 48]. Both the glycocalyx and adherens junctions are modified by stimuli such as flow and growth factors, and these changes vary over the course of days [49–51]. It is therefore possible that the peak and then decay in cell-associated vitronectin relate to changes in the glycocalyx and adherens junctions that affect transmembrane transport. Additional experiments are needed to test these hypotheses, as well as to determine how vitronectin transport is affected by flow and growth factors in vivo.

The differences in cell-associated vitronectin in endothelial monolayers exposed to steady laminar and oscillating disturbed flow also have important implications for vascular function. Cell-associated vitronectin increased plasminogen system activity, which is important in hemostasis, coagulation, vascular remodelling, and angiogenesis. Multimeric vitronectin binds and stabilizes PAI-1 in its active form, thereby decreasing plasminogen system activity [52, 53]. Thus, the *α*_v_*β*_5_ integrin-mediated increase in cell-associated vitronectin would decrease active PAI-1 and in turn increase plasminogen system activity. Endothelial cells exposed to steady laminar flow show the highest cell-associated vitronectin, and this may be important in the way that fluid forces control coagulation, neointimal formation, and endothelial sprouting [54–56].

In conclusion, our data show that shear stress and FGF-2 increase cell-associated vitronectin through *α*_v_*β*_5_ integrin activation and that this then increases plasminogen system activity. These data highlight the complex, multifaceted actions of mechanical stimuli and growth factors in vascular remodelling and elucidate how their effects may be altered in diseases in which vitronectin levels are varied, such as cancer, infarction, and atherosclerosis. In clinical therapies focused on vascular remodelling, treatment decisions should take into account the local biomechanical and biochemical stimuli as well as the extracellular matrix content, as all of these have significant interrelated effects on endothelial cell functions.

## Abbreviations

BBmVEC: Bovine brain microvascular endothelial cells
DMEM: Dulbecco's modification of Eagle's medium
EBM-2: Endothelial basal medium-2
EGM-2: Endothelial growth medium-2
FBS: Fetal bovine serum
FGF-2: Fibroblast growth factor-2
HUVEC: Human umbilical vein endothelial cells
mVTN: Multimeric vitronectin
nVTN: Native vitronectin
PAEC: Porcine aortic endothelial cells
PAI-1: Plasminogen activator inhibitor-1
PSMC: Porcine smooth muscle cells
uPA: Urokinase plasminogen activator
uPAR: Urokinase plasminogen activator receptor
VEGF: Vascular endothelial growth factor.
